# A Narrative Review on Neurorehabilitation in India: Current Scenario, Unmet Needs, and the Road to the Future

**DOI:** 10.7759/cureus.105698

**Published:** 2026-03-23

**Authors:** Ajay Emani, Ramakant Yadav, Midhun Mohan

**Affiliations:** 1 Neurology, Uttar Pradesh University of Medical Sciences, Etawah, IND

**Keywords:** access to care, assistive technology, community-based rehabilitation, india, neurorehabilitation, spinal cord injury, stroke rehabilitation, telerehabilitation, traumatic brain injury, workforce

## Abstract

Neurorehabilitation is crucial for improving function and participation after neurological disorders. In India, rising survival has increased rehabilitation needs, but services remain fragmented and unevenly distributed. The objective of this study was to summarize the current status of neurorehabilitation in India, including service delivery, access, workforce, technology use, and system-level challenges. Hence, a narrative review of the literature (2010-2025) from major databases and policy sources was synthesized thematically across predefined domains. It was found that there is a substantial unmet need for neurorehabilitation in India, with poor post-discharge continuity, underutilization (especially in rural areas), and workforce shortages. While structured inpatient rehabilitation improves outcomes, its reach is limited. Tele-neurorehabilitation and mHealth show promise but remain unevenly implemented. Key barriers include affordability, accessibility, and lack of standardized outcome measures. Thereafter, it was concluded that neurorehabilitation in India faces high demand but fragmented delivery and inequitable access. Strengthening care pathways, expanding and training the workforce, leveraging hybrid telerehabilitation models, and standardizing outcome measures are essential priorities.

## Introduction and background

Neurorehabilitation is a cornerstone of recovery for people living with the sequelae of stroke, spinal cord injury (SCI), traumatic brain injury, neurodegenerative disorders, and paediatric neurodisability, with goals that extend beyond impairment reduction to independence, participation, and quality of life [1,2]. In India, the growing survival after acute neurological and traumatic events has expanded the population living with long-term disability, increasing the demand for sustained, multidisciplinary rehabilitation across the life course [3,4]. However, access to structured neurorehabilitation remains uneven, shaped by urban concentration of services, variable team availability, financial barriers, and the built-environment challenges that determine whether functional gains translate into real-world participation [5].

Despite increasing attention to rehabilitation and disability inclusion, the Indian neurorehabilitation ecosystem is characterized by fragmentation across the continuum of care. Many patients experience a "post-discharge cliff," where acute hospital care is not systematically connected to post-acute therapy, follow-up, or community reintegration support, and families [6-8] often become the default long-term providers of care [9]. The available evidence also suggests that, while organized inpatient neurorehabilitation can yield meaningful functional and cognitive gains, scaling these benefits nationally is constrained by workforce shortages, limited multidisciplinary teams, and inconsistent outcome measurement that hampers benchmarking and quality improvement [10-13].

This narrative review synthesizes evidence published between 2010 and 2025 to describe the current scenario of neurorehabilitation in India, spanning disease burden, service delivery pathways, workforce and training capacity, condition-specific evidence, technology-enabled models, policy and financing context, barriers to access, and outcome measurement gaps. We aim to provide clinicians, administrators, and policymakers with an India-specific overview of what is currently known, where the major bottlenecks lie, and which pragmatic, equity-oriented strategies - such as strengthened discharge-to-community continuity, supervised task-sharing, hybrid telerehabilitation, and standardized outcome datasets - may accelerate progress toward accessible, high-quality neurorehabilitation at scale.

## Review

Metholodogy

Search Strategy and Sources

This narrative review was conducted to summarize the current scenario of neurorehabilitation in India, with an emphasis on service delivery, access, workforce, and evidence from common neurological conditions. A structured but non-systematic literature search was performed across major biomedical and multidisciplinary databases, primarily PubMed/MEDLINE, Scopus, and Google Scholar, supplemented by IndMED to capture Indian-indexed journals and reports that may be under-represented in international databases. Where relevant and accessible, selected Cochrane resources were consulted for high-level evidence or background context. In addition, key government and non-governmental organization (NGO) documents (e.g., policy statements, disability and rehabilitation program reports, guidance documents) were reviewed to contextualize the literature within the realities of the Indian health system and disability policy.

Searches were performed using combinations of controlled vocabulary (where available) and free-text terms related to neurorehabilitation and India. Core search phrases included “neurorehabilitation India”, “neurological rehabilitation India”, “rehabilitation services India”, “stroke rehabilitation India”, “spinal cord injury rehabilitation India”, “traumatic brain injury rehabilitation India”, “telerehabilitation India”, “tele-neurorehabilitation India”, “community-based rehabilitation India”, “rehabilitation workforce India”, “physiatry India”, “occupational therapy India”, and “speech therapy India.” These were iteratively refined using Boolean operators and database-specific filters to identify additional condition-specific and systems-focused literature (e.g., access, barriers, workforce, outcome measures, program evaluations). The primary timeframe of interest was 2010-2025, showing the contemporary developments in Indian neurorehabilitation; however, selected older papers and policy documents were included when they provided foundational context for service organization or disability policy.

Inclusion and Exclusion Approaches

Because this is a narrative review, we used an inclusion approach designed to maximize relevance and breadth rather than restrict to a narrow study design hierarchy. We included peer-reviewed publications and selected grey literature that met one or more of the following criteria: (i) conducted in Indian settings or explicitly reporting India-specific data; (ii) describing neurorehabilitation service delivery models, pathways, or systems organization (public, private, NGO, community, home-based); (iii) reporting on access, utilization, barriers, or inequities relevant to neurorehabilitation; (iv) addressing workforce, training or multidisciplinary team capacity; (v) condition-specific neurorehabilitation evidence for high-burden conditions (e.g., stroke, SCI, acquired brain injury/traumatic brain injury, paediatric neurodisability, and selected neurodegenerative conditions); (vi) program evaluations, audits, surveys, registries, or implementation/feasibility studies (including telerehabilitation and technology-enabled models); and (vii) Indian policy documents, guidance, and reports relevant to disability and rehabilitation.

We excluded papers that were not relevant to rehabilitation (e.g., purely acute management or surgical/procedural studies without a rehabilitation component) and papers from non-Indian settings unless they were used briefly for comparison or to provide general background when India-specific evidence was limited. Publications outside the target timeframe were generally excluded, except when an older source was necessary to frame a historically important policy or service-development milestone.

Data Extraction and Thematic Synthesis

Titles, abstracts, and full texts (where available) were screened for relevance to the review objectives. For included sources, key information was extracted into a working evidence table capturing: publication year, setting (e.g., tertiary/quaternary centre, district facility, community), population/condition, study type (e.g., survey, cohort, randomized trial, qualitative study, scoping review, policy/report), intervention or service model (if applicable), and main findings relevant to neurorehabilitation access, delivery, outcomes, or system constraints. Given the heterogeneity of evidence (designs, settings, outcomes, and reporting), a meta-analysis was not planned.

Findings were synthesized using a thematic narrative approach. Evidence was organized into pre-specified themes aligned with the manuscript structure: burden and rehabilitation need; current service delivery pathways and settings; access and equity; workforce and training capacity; condition-specific neurorehabilitation (stroke, SCI, acquired brain injury/traumatic brain injury, paediatric neurodisability, and selected conditions where available); technology-enabled and emerging models (tele-neurorehabilitation, mHealth); policy and systems context; barriers across patient, provider, and system levels; outcome measurement and data gaps; and pragmatic recommendations for India. Within each theme, findings were summarized descriptively, triangulating across study types where possible, and highlighting consistencies, divergences, and key gaps in the evidence base.

**Figure 1:**
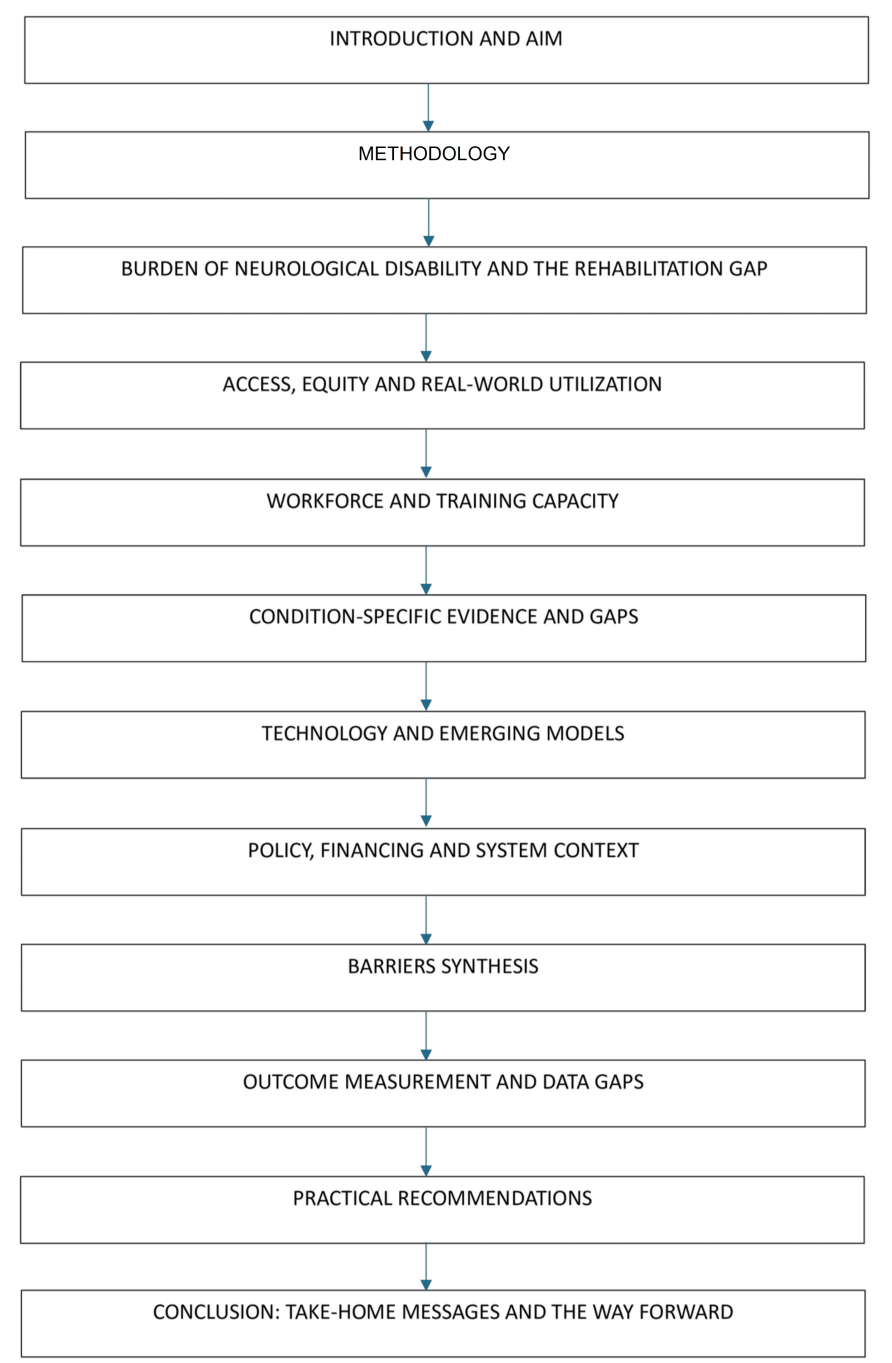
Navigation map for this narrative review Image credits: Ajay Emani. Generated in MS Word (Microsoft® Corp., Redmond, WA) using Smart Art

Figure 1 presents the navigation map for this review.

Burden of neurological disability and rehabilitation needs in India

National Disability Burden and "Rehabilitation Gap"

India's disability burden is substantial at a population level, with nationally representative data (NSS 2018) estimating an overall disability prevalence of 2.2%, translating to ~26.8 million persons with disabilities (PWD) in absolute numbers. Beyond prevalence, the same dataset highlights a large “rehabilitation and support gap,” in which public support systems and enabling environments do not effectively reach the majority of PWDs [14].

At a systems level, structural barriers that directly limit rehabilitation participation are common, including difficulty using public transport (~40%) and difficulty accessing public buildings (~57.7%). Disability also has major livelihood consequences, with ~60.7% of those who had employment losing their job after disability onset, which can worsen the affordability of long-term neurorehabilitation. A further barrier to accessing entitlements is documentation, as ~69.6% reportedly did not have a disability certificate, potentially excluding many from concessions and government schemes that could indirectly support rehabilitation access [14].

Stroke as a Leading Driver of Adult Neurodisability

Stroke is repeatedly identified as a major contributor to disability in India, with reported prevalence ranging from ~84-262 per 100,000 in rural areas to ~334-424 per 100,000 in urban areas, and incidence ~119-145 per 100,000/year (as summarized in an India-focused review of stroke epidemiology and services). Despite the scale of stroke-related disability, stroke rehabilitation services have historically been described as underdeveloped, largely due to a shortage of trained personnel and concentration of services in urban/private settings [15].

At the household level, post-discharge needs appear near-universal, with one mixed-methods study in an urban Indian setting reporting that 100% of stroke survivors and caregivers reported rehabilitation needs, and none felt all needs were met. Information and education needs were especially prominent (approximately 82% of survivors and 92% of caregivers reporting substantial unmet information needs), suggesting that even basic navigation of recovery and services is a major gap [16].

Financial needs were also common (about 70% of survivors and 75% of caregivers reported significant financial needs), indicating how out-of-pocket rehabilitation costs can act as a barrier even in metropolitan contexts [16]. Utilization data from a tertiary follow-up sample showed that roughly one-third of stroke survivors were not using any rehabilitation services despite disability, and rural patients had markedly lower utilization (~45.5%) than urban counterparts [17].

Among those not utilising services, common reasons included not knowing what services exist (despite knowing rehabilitation is needed) and lack of local availability, underscoring that both “demand-side” awareness and “supply-side” service distribution drive unmet need [17]. Finally, India's largest pragmatic stroke rehabilitation trial (family-led model) did not reduce death/dependency compared with usual care, cautioning that caregiver-only task shifting may be insufficient as a stand-alone solution where professional/community rehabilitation capacity is limited [18].

SCI and Long-Term Participation Restrictions

SCI contributes to severe, long-duration disability with high participation restrictions, particularly once patients return to non-accessible community environments [19]. A rural South India follow-up study reported that community reintegration outcomes commonly declined by one year after discharge despite completion of inpatient rehabilitation, emphasising that gains achieved in hospitals may not be sustained without environmental supports and follow-up systems [19].

Key barriers to participation included architectural/environmental barriers, poor socioeconomic status, and comorbidities that limit mobility and community engagement [19]. More recent Indian data on chronic SCI similarly highlight persistent environmental and financial constraints, including inaccessible basic amenities and heavy dependence on out-of-pocket spending or non-governmental support. Long-term vocational impact appears profound, with substantial drops in employment after injury and limited insurance coverage for rehabilitation and ongoing care [20].

Acquired Brain Injury and the “Effectiveness-Access” Mismatch

Evidence from an Indian inpatient neurorehabilitation cohort suggests that structured multidisciplinary rehabilitation can produce meaningful gains in functional independence and cognition over short inpatient stays, with improvements maintained at follow-up [21]. However, the same work explicitly frames these improvements against a broader context where many acquired brain injury survivors in India do not receive comprehensive rehabilitation and remain with long-term disability that burdens families. This creates an “effectiveness-access mismatch” in which benefit is demonstrable among those who reach services, but population impact remains constrained by limited coverage [21].

**Figure 2:**
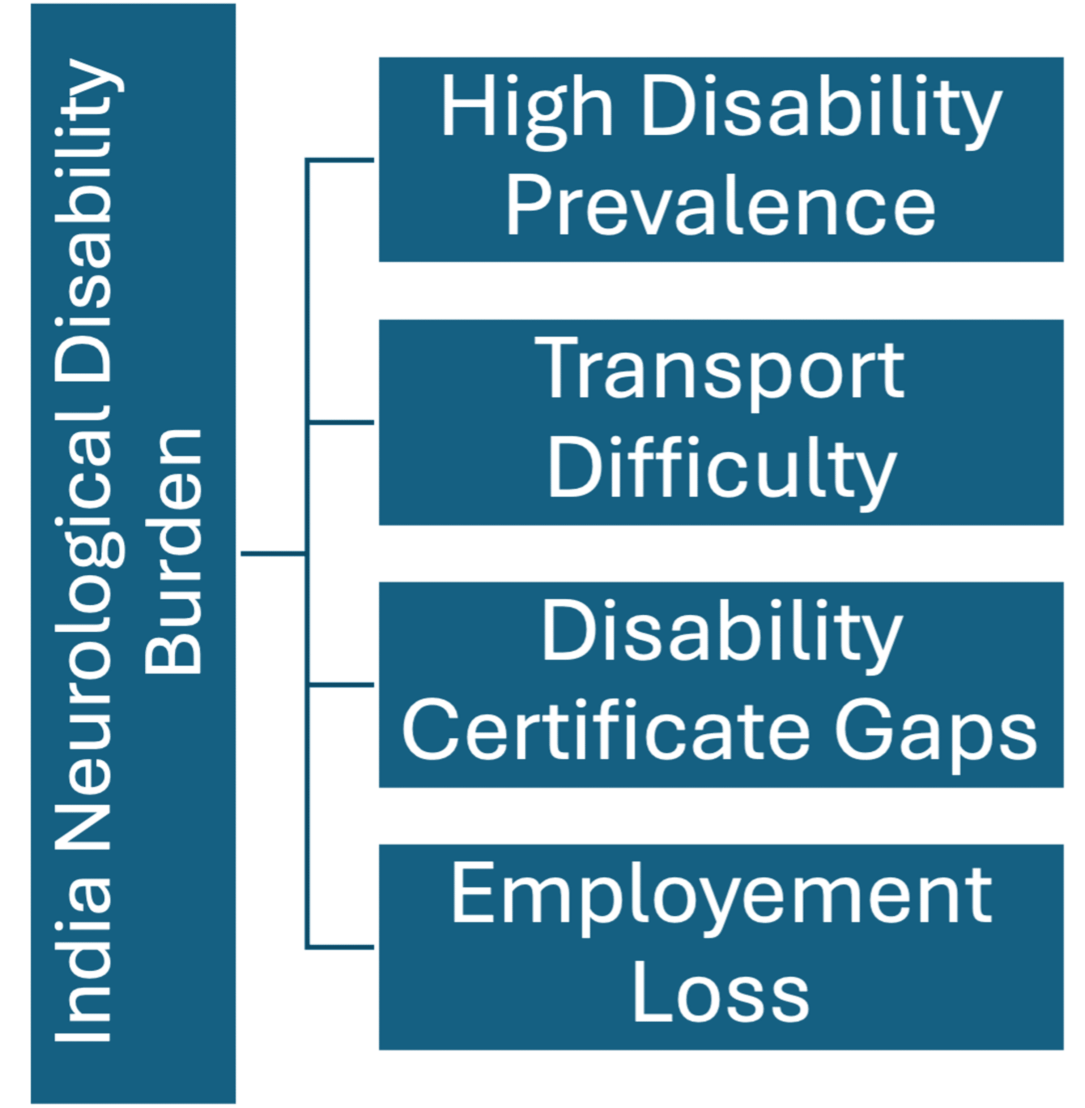
Burden of neurological disability and rehabilitation need in India

Figure 2 shows the burden of neurological disability and rehabilitation needs in India.

Rural Context and Persistent Barriers (Affordability, Awareness, Accessibility)

Community-level data from rural Uttar Pradesh (early 2010s) documented a high burden of long-term neurological impairment and quantified barriers to rehabilitation use, with financial constraints (44%) and lack of awareness (43%) as leading reasons for not receiving or discontinuing rehabilitation [22]. While the diagnostic mix in that dataset reflects historical patterns (including poliomyelitis), the barrier profile, cost, awareness gaps, and inaccessible environments align closely with later national disability findings on transport/building access barriers [22].

Expert commentary from Indian rehabilitation leadership has similarly emphasized that improved survival after neurological insults increases the pool of people living with chronic deficits. In contrast, rehabilitation facilities remain limited outside a few institutions, particularly affecting rural areas [23].

Summary

What the burden implies for this review: Taken together, available Indian evidence suggests that neurological conditions (especially stroke, SCI, and acquired brain injury) generate large and sustained rehabilitation needs, while service reach is limited by workforce and infrastructure constraints, high out-of-pocket costs, and barriers in transport/buildings that restrict participation. This burden and gap framing provides the rationale for examining the current scenario of neurorehabilitation services, workforce, models of care, technology use, and policy implementation in India in the subsequent sections [23].

Current Neurorehabilitation Service Delivery in India

Care continuum and referral pathways (acute → post-acute → community): In India, the neurorehabilitation "continuum of care" is frequently fragmented, with many patients transitioning from acute care to home without a structured pathway for therapy, follow-up, or community reintegration, as highlighted by Pandian et al. [15]. Mixed-method evidence from an urban cohort of stroke survivors shows that post-discharge rehabilitation needs are near-universal, yet the system-level linkage to services is weak, as described by Kamalakannan et al. [16].

A central pathway failure is informational discontinuity - patients and caregivers often leave the hospital without clear education on recovery, home programs, or where to access services, contributing to downstream underutilization, as reported by Kamalakannan et al. [16].

This “navigation gap” is reinforced by facility-level observations where survivors cite not knowing what services exist or how to access them, even when they accept rehabilitation as necessary, as documented by Mahak et al. [17]. The ATTEND multicentre RCT also indirectly illustrates the typical pathway context in India - "usual care" often comprises limited formal rehabilitation after discharge - within which a structured family-led program did not improve death/dependency outcomes, as shown by the ATTEND Collaborative Group [18]. Taken together, the current scenario suggests that the dominant model remains “family-supported recovery with variable professional input,” rather than a reliably integrated, staged neurorehabilitation pathway, as interpreted by the ATTEND Collaborative Group [18].

Beyond stroke, a similar transition problem is seen in SCI contexts where timely entry into rehabilitation can be strongly influenced by whether structured counselling and guidance occur during the acute phase, as demonstrated by Aiyer et al. [24].

In that cohort, early counselling was associated with much earlier initiation of rehabilitation compared with those who lacked such guidance, supporting counselling/navigation as a practical “bridge” between acute services and rehabilitation uptake, as reported by Aiyer et al. [24].

Service delivery settings and prevailing models of care

Neurorehabilitation in India is delivered through a patchwork of tertiary/quaternary academic centres, private hospitals and clinics, smaller rehabilitation units, and limited community-facing models, with substantial variation in team composition and available interventions, as summarized by Surya et al. [25]. A national survey of professionals suggested that even pre-pandemic, a sizeable proportion of practice occurred outside strong multidisciplinary teams. Key specialist roles were absent in many units, reflecting uneven service maturity across settings, as described by Surya et al. [25]. The same survey also indicates that advanced technologies are present in a minority of units, while basic physiotherapy modalities dominate, implying that “what neurorehabilitation means in practice” often remains low-technology and discipline-limited in many sites [25].

Where comprehensive inpatient neurorehabilitation exists - typically in high-end tertiary/quaternary centres - measurable functional and cognitive gains have been demonstrated in acquired brain injury cohorts, underscoring what organized services can achieve when accessible, as shown by Patil et al. [21]. However, such models are not widely available, and the “centre-of-excellence” pattern contributes to geographic concentration of expertise, with limited downstream coverage for district-level follow-up, as inferred from Kamalakannan et al. [16].

During the COVID-19 period, services were disrupted nationwide, and many centres shifted toward remote follow-ups and telerehabilitation as a pragmatic alternative rather than an optional innovation, as documented by Surya et al. [25]. In parallel, India-specific demonstrations of tele-neurorehabilitation feasibility-linking quaternary teams to district hospitals-suggest a viable service-delivery “extension model” for underserved regions, as reported by Khanna et al. [26].

Access, equity, and real-world utilization (urban-rural, financial, environmental)

Utilization data from a tertiary follow-up cohort indicate that a substantial minority of stroke survivors do not use any rehabilitation services despite disability, pointing to a consistent access-uptake gap, as shown by Mahak et al. [17]. The same study highlights a marked urban-rural disparity in utilization, with rural survivors far less likely to receive rehabilitation than urban counterparts, reflecting structural access limitations rather than merely preference, as reported by Mahak et al. [17]. When reasons for non-utilization are elicited, knowledge gaps (not knowing where to go) and local non-availability of services emerge prominently, indicating both “demand-side” and “supply-side” barriers, as documented by Mahak et al. [17].

Rural community data (though from an earlier epidemiologic context) demonstrate that financial constraints and lack of awareness are dominant barriers to access to neurorehabilitation, alongside transport and environmental inaccessibility, as reported by Kumar et al. [22]. Expert commentary aligns with these findings, emphasising that architectural barriers and limited awareness can be as disabling as the neurological impairment itself in rural contexts, as argued by Baruah et al. [23].

Nationally representative evidence further quantifies these structural constraints: large proportions of PWD report difficulty using public transport and accessing public buildings, which directly constrains participation and the feasibility of repeated therapy visits, as reported by Mirza et al. [14]. The same analysis indicates major gaps in receipt of government support and documentation (e.g., disability certification), suggesting that entitlements intended to enable rehabilitation and inclusion often do not reach the majority of eligible individuals, as shown by Mirza et al. [14].

Condition-specific long-term follow-up in SCI illustrates how environmental and socioeconomic barriers can erode functional gains after discharge, with mobility and infrastructure barriers limiting community reintegration for rural survivors, as reported by Sekaran et al. [19]. Contemporary qualitative evidence similarly describes persistent gaps in accessible housing, sanitation, transport, and financing for long-term SCI survivors, reinforcing that rehabilitation outcomes in India are tightly coupled to social determinants and built environment constraints, as shown by Mullerpatan et al. [20].

Finally, technology-enabled approaches (telemedicine and mHealth) are increasingly positioned as equity tools to reduce travel burden and extend specialist input, with feasibility demonstrated both via tele-neurorehabilitation hub-and-spoke models and smartphone-based home guidance, as reported by Khanna et al. and Sureshkumar et al. [26,27].

Workforce and training capacity for neurorehabilitation in India

India's neurorehabilitation capacity is constrained not only by the absolute number of trained professionals but also by uneven team composition across facilities [15]. A nationwide survey during the COVID-19 period provides a systems-level snapshot showing that many rehabilitation units lacked key specialist roles and that service delivery was disrupted across settings. The same survey also reported a strong perceived need for additional neurorehabilitation training among professionals, suggesting both a skills gap and readiness for structured upskilling initiatives. These findings imply that “multidisciplinary neurorehabilitation” is often an aspirational model rather than consistently available routine practice, particularly outside major tertiary centres [15].

A second layer of the workforce challenge is the heavy reliance on families and community workers as de facto providers of long-term rehabilitation support [18,23]. A comprehensive training needs assessment among community-based workers and caregivers in India identified specific skill gaps in managing spasticity, assistive devices, home-based strategies, and context-specific counselling, highlighting that task-sharing requires structured competency-based training rather than informal transfer of responsibility [28]. This evidence supports a scalable “middle-tier” approach in which rehabilitation assistants or community-based workers deliver structured components of care under supervision, with escalation pathways [23,28]. Overall, strengthening workforce capacity in India likely requires both specialist expansion and deliberate development of supervised community-linked rehabilitation cadres [25,28].

Condition-specific neurorehabilitation: Indian evidence and observed gaps

Stroke Neurorehabilitation

Stroke contributes substantially to disability burden in India and generates large post-acute rehabilitation needs [15]. Mixed-method evidence from an urban cohort found that 100% of stroke survivors and caregivers reported rehabilitation needs after discharge and none felt all needs were met [16]. Information and education needs were especially prominent, with high proportions of survivors and caregivers reporting substantial unmet information needs that directly affect home-based recovery and service navigation [16]. Utilization data from a tertiary follow-up cohort showed that roughly one-third of stroke survivors were not using any rehabilitation services despite disability [17]. The same study demonstrated marked urban-rural disparity in utilization, suggesting that geographic access is a major determinant of therapy exposure in routine practice [17].

The strongest India-specific trial evidence testing a pragmatic model is the ATTEND multicentre RCT of family-led stroke rehabilitation [18]. ATTEND found no improvement in death or dependency compared with usual care, indicating that caregiver-only delivery of structured exercises is insufficient as a standalone solution at scale [18]. These findings support a shift toward hybrid models that combine caregiver enablement with professional or paraprofessional inputs and structured follow-up systems [16,18].

SCI Neurorehabilitation

SCI in India illustrates the long-horizon nature of neurorehabilitation needs and the dependence of outcomes on environment and social systems [19]. Follow-up data from rural South India demonstrated that community reintegration can deteriorate after discharge even when inpatient rehabilitation was received, reflecting the impact of real-world barriers on participation outcomes [19]. More recent Indian evidence from long-term SCI survivors describes a persistent lack of accessible amenities, inaccessible transport and public spaces, unemployment, and substantial out-of-pocket financing for ongoing care. These findings indicate that durable SCI outcomes require rehabilitation systems that integrate community accessibility, assistive technologies, vocational pathways, and financing mechanisms beyond the inpatient phase [19].

A practical service-delivery lever is acute-phase counselling and navigation support [24]. In a prospective cohort, early counselling during acute hospitalization was associated with substantially earlier initiation of rehabilitation compared with patients who did not receive structured counselling [24]. This suggests that systematic discharge planning and counselling can partially mitigate delays to rehabilitation even before large-scale infrastructure expansion [24].

Acquired Brain Injury and Traumatic Brain Injury

Indian inpatient neurorehabilitation cohorts suggest meaningful gains in cognition and functional independence during structured inpatient rehabilitation programs [21].

These improvements provide supportive evidence for the effectiveness of organized multidisciplinary rehabilitation when patients can access it [21]. However, the broader implication is an “effectiveness-access mismatch”, where demonstrated benefit exists in those reaching services but coverage remains limited in many regions [21,23].

Paediatric Neurorehabilitation

Paediatric rehabilitation in India has expanded through institutional growth and programmatic initiatives but remains unevenly accessible, particularly for rural families [11].

Expert synthesis highlights persistent rural shortages of paediatric-trained therapists, continued reliance on NGOs and parent networks, and the need for community-oriented models for sustained therapy [11,29,30]. The paediatric context reinforces that long-term neurorehabilitation depends on family support systems, inclusive education, and accessible community environments in addition to clinical therapy [11,23].

Technology and emerging models in India

Tele-neurorehabilitation is increasingly positioned as a pragmatic access-extender in India because it can link specialist expertise to district-level settings [26]. A quaternary-centre experience demonstrated that tele-neurorehabilitation could support management of many cases locally with remote specialist guidance while triaging a subset for higher-level referral [26]. This model is particularly relevant given the urban concentration of rehabilitation expertise and travel-related barriers to repeated therapy visits [26].

The COVID-19 period accelerated telerehabilitation adoption across India, with national survey data indicating widespread uptake during service disruptions [31]. This rapid pivot suggests operational feasibility when necessity forces change, although it does not by itself establish long-term effectiveness or equity.

Mobile health tools represent an additional “low-cost scalable layer” for home-based rehabilitation education [27]. A feasibility pilot of the “Care for Stroke” smartphone-enabled educational intervention reported high acceptability, usability with minimal training, and caregiver empowerment in semi-urban and rural Indian settings [27]. Given evidence that post-stroke information needs are a major unmet domain in India, mHealth education tools can be conceptualized as an intervention targeting the navigation and knowledge gap rather than replacing therapist-delivered care [16,27].

Policy, financing, and systems context relevant to neurorehabilitation

Nationally representative evidence indicates that disability in India is common in absolute numbers and that access to supports and enabling environments remains limited. Large proportions of persons with disabilities report difficulty using public transport and accessing public buildings, which directly constrains rehabilitation participation and community reintegration. The same analysis reports high rates of employment loss after disability onset, highlighting the importance of vocational outcomes and social protection as rehabilitation endpoints. Administrative barriers also matter, as a large majority did not possess disability certificates in the dataset, which may limit access to entitlements that can indirectly support rehabilitation.

Financing constraints are strongly visible in long-term SCI experiences in India, where ongoing care and rehabilitation are predominantly funded out-of-pocket with limited insurance coverage. Qualitative findings from long-term SCI survivors emphasize that lack of accessible infrastructure and limited financial protection impede “respectful integration” and sustained functioning despite initial rehabilitation. These data support framing neurorehabilitation as an intersectoral policy issue spanning health systems, disability rights implementation, financing protection, and accessible infrastructure. 

Barriers to effective neurorehabilitation in India

Patient and family barriers include major information gaps, financial constraints, and a lack of structured guidance after discharge [16]. In a tertiary follow-up cohort, many stroke survivors who did not utilize rehabilitation cited not knowing where to access services and local unavailability. This indicated that navigation failure and supply limitations coexist [17]. Rural community data further identify financial constraints and lack of awareness as leading barriers to rehabilitation uptake, suggesting persistent inequity beyond tertiary networks [22].

Provider and service barriers include uneven multidisciplinary staffing and variable service intensity across facilities. Pandemic-era survey data additionally show how quickly therapy intensity and direct supervision can be disrupted, revealing fragility in service delivery when staffing and operational constraints tighten [25].

System and environmental barriers include transport and built-environment inaccessibility, which limit the feasibility of repeated rehabilitation visits and restrict community participation. Expert commentary emphasizes that architectural barriers and low awareness in rural areas can be as limiting as medical impairment, supporting the need for community-based rehabilitation and environmental modifications [23]. Condition-specific evidence in SCI shows that environmental and socioeconomic barriers can erode gains achieved during inpatient rehabilitation and constrain reintegration over the long term [19].

Outcomes measurement, benchmarking, and data gaps

A key limitation in Indian neurorehabilitation research is heterogeneity in outcome measurement, which reduces comparability across studies and weakens the case for benchmarking [10]. A scoping review of stroke rehabilitation research in India identified a large number of outcome measures used across studies and highlighted that very few measures were developed or culturally validated in India [10]. The same review noted that Indian stroke rehabilitation research has often emphasized impairment and basic ADL outcomes while under-measuring participation, psychosocial outcomes, and caregiver domains [10].

Indian SCI research demonstrates the feasibility and relevance of participation-focused metrics in rural contexts, supporting the argument that outcomes should extend beyond impairment to reintegration and environmental barriers [19]. Inpatient ABI cohorts using standardized metrics such as FIM and MoCA provide examples of measurable functional and cognitive change during rehabilitation, which can inform routine outcome capture models [21].

Synthesized together, these studies support the adoption of a pragmatic minimum dataset for Indian neurorehabilitation that captures function, participation, caregiver burden, and context-sensitive quality-of-life domains [10,19,21].

Practical recommendations for India

Short-Term Priorities (0-2 Years)

Implement standardized discharge counselling and referral navigation for stroke and SCI to reduce delays and drop-off after acute care [16,24]. Embed patient and caregiver education as a routine discharge deliverable because information gaps are a dominant unmet need in Indian stroke survivors and caregivers [16]. Avoid positioning caregiver-only models as the default national solution, since family-led rehabilitation did not improve death/dependency at scale in the ATTEND trial [18]. Adopt hybrid follow-up pathways using teleconsultation for review and goal-setting, particularly to support district-level teams and reduce travel barriers [26].

Medium-Term Priorities (3-5 Years)

Scale hub-and-spoke neurorehabilitation models linking tertiary hubs to district hospitals and community providers using tele-neurorehabilitation workflows [25,26]. Develop competency-based training programs for community-based workers and caregivers to support supervised task-sharing and reduce dependence on specialist scarcity [23,28]. Integrate mHealth education tools into post-discharge stroke pathways to address information gaps and support structured home programs where therapist access is limited [16,27]. Begin routine collection of a minimum outcome dataset across rehabilitation programs to enable quality improvement and comparability across settings [10,21].

Long-Term Priorities (5-10 Years)

Strengthen financing protection for long-term rehabilitation and assistive needs because Indian SCI survivors report high out-of-pocket costs with limited insurance coverage [20]. Invest in accessible transport and barrier-free public infrastructure since national data show pervasive access limitations that constrain rehabilitation participation [23]. Prioritize participation and vocational reintegration as core rehabilitation outcomes, supported by evidence of employment loss after disability and persistent unemployment after SCI. Standardize and culturally validate outcome measures for India to strengthen the evidence base, support registries, and guide policy and reimbursement decisions [10].

Research gaps and future directions: Key evidence gaps remain across the neurorehabilitation continuum in India, particularly in areas that directly influence scalability and policy uptake. There is a need for cost-effectiveness research that compares therapy “dose” and models of care (facility-based vs home-/community-based, and hybrid approaches) and evaluates the value proposition of technology-enabled rehabilitation (telerehabilitation, mHealth, and advanced devices) in different resource settings. Pragmatic, real-world trials are especially required in district and rural contexts, where workforce constraints, travel burden, and service availability differ substantially from tertiary centres. Implementation science should be prioritized to identify which delivery models reliably scale (e.g., hub-and-spoke networks, supervised task-sharing, community-based rehabilitation) and what enabling factors (training, financing, referral systems, digital workflows) drive sustained adoption. More evidence is also needed on caregiver-focused interventions, including caregiver training models that do not simply shift burden, and caregiver mental health supports to reduce burnout and improve continuity of home-based rehabilitation. Future studies should systematically capture long-term outcomes, including participation, community reintegration, and return-to-work metrics - as these outcomes best reflect whether neurorehabilitation is translating into meaningful social and economic recovery in Indian settings.

Limitations of this narrative review

This review is narrative rather than systematic, and while the search was structured and transparent, it does not provide exhaustive retrieval or formal risk-of-bias grading across all included sources. The included evidence is heterogeneous in study design, populations, outcomes, and reporting standards, limiting direct comparison and precluding quantitative synthesis. Publication patterns may bias available literature toward tertiary and urban centres, where services and documentation are more established, potentially underrepresenting district-, rural-, and community-based care. In several domains, high-quality comparative Indian studies remain limited, particularly regarding the long-term effectiveness of different service models, economic evaluation, and implementation outcomes, which constrains the strength of inferences that can be drawn about “best” approaches for scale-up.

## Conclusions

The current neurorehabilitation scenario in India is characterized by substantial and growing need alongside fragmented service delivery and inequitable access, with a marked “drop-off” after hospital discharge and strong dependence on families for long-term support. While centres with structured multidisciplinary rehabilitation demonstrate meaningful functional gains, these services are unevenly distributed, and real-world utilization remains constrained by workforce shortages, affordability, limited local availability, and environmental barriers that restrict participation. Emerging technology-enabled models, including telerehabilitation and mHealth-supported home programs, show feasibility and provide promising pathways to extend reach, but require careful integration into care pathways and attention to equity, quality, and outcomes measurement.

Strengthening neurorehabilitation in India will require a focused set of priorities: building reliable discharge-to-community continuity through standardized counselling and referral pathways; expanding workforce capacity through competency-based training, supervised task-sharing, and improved multidisciplinary coverage; and scaling hybrid hub-and-spoke models that link tertiary expertise with district-level delivery. In parallel, policy and financing reforms should reduce out-of-pocket burden and promote accessibility in transport and public infrastructure so that rehabilitation gains translate into real-world participation. Finally, routine adoption of a minimum standardized outcomes dataset - including functional, participation, caregiver, and return-to-work metrics - will be essential to benchmark quality, support registries, and guide investment toward scalable, evidence-informed neurorehabilitation models.
